# Diagnostic accuracy in NSCLC lymph node staging with Total-Body and conventional PET/CT

**DOI:** 10.1007/s00259-025-07177-3

**Published:** 2025-03-21

**Authors:** Clemens Mingels, Mohammad H. Madani, Fatma Sen, Hande Nalbant, Jonathan W. Riess, Yasser G. Abdelhafez, Ahmadreza Ghasemiesfe, Axel Rominger, Michele Guindani, Ramsey D. Badawi, Benjamin A. Spencer, Lorenzo Nardo

**Affiliations:** 1 Department of Radiology, University of California Davis, Sacramento, CA USA; 2 Department of Nuclear Medicine, Inselspital, Bern University Hospital, University of Bern, Bern, Switzerland; 3 Division of Hematology/Oncology, Department of Internal Medicine, University of California Davis, Sacramento, CA USA; 4 Nuclear Medicine Unit, South Egypt Cancer Institute, Assiut University, Assiut, Egypt; 5 Department of Biostatistics, University of California Los Angeles, Los Angeles, CA USA; 6 Department of Biomedical Engineering, University of California Davis, Davis, CA USA

**Keywords:** Total-body PET, Lung cancer, SAFOV PET, [^18^F]FDG, N-staging

## Abstract

**Introduction:**

Our aim was to characterize the diagnostic accuracy indices for nodal (N)-staging with [^18^F]FDG Total-Body (TB) and short-axial field-of-view (SAFOV) PET/CT in non-small cell lung cancer (NSCLC) patients referred for staging or restaging.

**Methods:**

In this prospective single center cross-over head-to-head comparative study 48 patients underwent [^18^F]FDG TB and SAFOV PET/CT on the same day. In total 700 lymph node levels (1R/L, 2R/L, 3a/p, 4R/L, 5, 6, 7, 8R/L, 9R/L, 10-14R/L) of 28 patients could be correlated to a composite reference standard (histopathological correlation, imaging after localized or systemic treatment), which allowed determination of true positive (TP), false positive (FP), true negative (TN) and false negative (FN) lesions. Lymph nodes were characterized semi-quantitatively by maximum standardized uptake value (SUV_max_), tumor-to-background ratio (TBR), metabolic tumor volume (MTV) and total lesion glycolysis (TLG) leading to threshold for each scanner.

**Results:**

TB and SAFOV PET/CT showed high diagnostic accuracy indices for patient-based N-staging. Sensitivity and specificity were 86.0% (CI: 77.0–95.0%) and 98.3% (CI: 97.3–99.3%) for TB; 77.2% (CI: 66.3–88.1%) and 97.4% (CI: 96.1–98.6%) for SAFOV PET. Positive predictive value was higher for TB (81.7%, CI: 71.9–91.5%) compared to SAFOV PET (72.1%, CI: 60.9–83.4%). However, this finding was not statistically significant (*p* = 0.08). Negative predictive values for TB (98.6%, CI: 97.9–99.6%) and SAFOV PET/CT (98.0%, CI: 96.9–99.1%) were comparable. Overall, NSCLC N-staging was affected in six cases on SAFOV and only in one case on TB PET/CT. Semi-quantitative analysis revealed a threshold of SUV_max_ 3.0 to detect TP lesions on both scanners. However, TBR, MTV and TLG thresholds were lower on TB compared to SAFOV PET (TBR: 1.2 vs. 1.7, MTV: 0.5 ml vs. 1.0 ml and TLG: 1.0 ml vs. 3.0 ml).

**Conclusion:**

TB and SAFOV PET/CT showed high diagnostic accuracy indices for N-staging in NSCLC patients. Sensitivity and PPV on TB PET/CT were slightly higher, compared to SAFOV PET/CT without statistical significance. However, TB PET/CT showed lower rate of incorrect N-staging and lower semi-quantitative thresholds for the detection positive mediastinal lymph nodes. Therefore, TB PET/CT might be advantageous in detecting small and low [^18^F]FDG-avidity mediastinal lymph node metastases in NSCLC patients.

**Supplementary Information:**

The online version contains supplementary material available at 10.1007/s00259-025-07177-3.

## Introduction

Lung cancer is the leading cause of cancer related death worldwide [1]. The most common type of lung cancer is non-small cell lung cancer (NSCLC) with its histologic subtypes e.g. adenocarcinoma, squamous cell carcinoma and large cell carcinoma [2]. [^18^F]FDG has been the tracer of interest to characterize tumor-(T)-stages, mediastinal lymph node metastases (N-stage) and distant metastases (M-stage) in PET/CT [3]. Therefore, [^18^F]FDG PET/CT is recommended in all major oncology guidelines for staging of NSCLC [4–6].

[^18^F]FDG PET/CT is especially recommended to help determine mediastinal lymph node involvement owing to its higher specificity (76–96%) compared to CT chest imaging (74–86%), which results in high negative predictive values (NPV) of 89–94% [7–9]. However, false positive results (FP) may occur in situations of granulomatous inflammations such as tuberculosis, sarcoidosis or sarcoid-like reactions after immunotherapy [10]. Therefore, National Comprehensive Cancer Network (NCCN) guidelines recommend histological correlation in cases of PET-positive lymph node findings [4]. Moreover, sensitivity is reported to be higher to detect mediastinal lymph node metastases (> 1 cm) for PET/CT (58–94%) compared to CT chest (51–64%) [7, 8]. Nevertheless, the sensitivity for mediastinal lymph node metastasis is not as high as its specificity e.g., due to the occurrence of low [^18^F]FDG-avidity lesions, which may lead to false negative (FN) PET interpretation [9].

The development of next-generation PET/CT scanners, including long-axial field-of-view and Total-Body (TB) PET/CT has changed the field of molecular imaging [11–13]. These scanners have proven to have superior signal collection efficiency and lower noise characteristics leading to higher lesion conspicuity in clinical routine over conventional short-axial field-of-view (SAFOV) PET-systems [14–18]. These superior characteristics enable imaging small structures with high precision even with low [^18^F]FDG uptake [11, 19–21]. This improvement may affect N-staging in NSCLC patients, especially in situations of small mediastinal lymph node metastases with low [^18^F]FDG uptake and could avoid FN PET interpretation.

In this prospective study we aimed to characterize the diagnostic accuracy indices for NSCLC N-staging on SAFOV and TB PET/CT in a head-to-head study design. Additionally, an exploratory aim was identifying semi-quantitative thresholds for true positive (TP) mediastinal lymph nodes on both scanners. We hypothesized that TB PET/CT could detect TP lesions with lower [^18^F]FDG uptake.

## Materials and methods

### Patient population, radiopharmaceutical and imaging study design

In this prospective cross-over single-center head-to-head comparative study 48 patients NSCLC were included for our analysis (male: 15, female: 33). Patients’ characteristics are outlined in Table 1.

**Table 1:** Patients’ characteristics for both short-axial field-of-view (SAOV) and Total-Body (TB) PET/CT

NSCLC subtypes (*n* = 48)	Sex	Age [years]	Study arm (1 = TB first, 2 = SAFOV first)
Adenocarcinoma (*n* = 30) Squamous cell carcinoma (*n* = 6)	Male (*n* = 15)	68	1 (*n* = 19)
Poorly differentiated carcinoma (*n* = 2) Not specified (*n* = 10)	Female (*n* = 33)	[54–84]	2 (*n* = 29)

Patients received a standard injection dose of 8.0 ± 20%mCi [^18^F]FDG intra-venously and were randomized into two different study arms. Study arm 1 included a research scan on the TB PET-system 57±10 min after injection of the radiopharmaceutical with a 22-minute acquisition in list-mode followed by a 22-minute SAFOV PET/CT scan at 92±10 min post injection [14]. Patients in study arm 2 received the 22-minute SAFOV PET/CT scan 62±14 min post injection first followed by the TB PET/CT research scan 91±17 min post injection.

### Reconstruction parameters and scanner characteristics

Patients were scanned on the uEXPLORER TB PET/CT scanner which has an axial FOV of 194 cm and spatial resolution of approximately 3.0 mm [11]. The uEXPLORER is equipped with a 160 slice CT scanner. Patients were imaged at 140 kVp and 50 mAs with automated dose modulation following the standard clinical protocol for PET/CT imaging [22]. TB PET images were reconstructed following the local clinical protocol with TOF enabled OSEM reconstruction algorithm, 2.344 mm^3^ isotropic voxel size, 4 iterations, 20 subsets, without post-reconstruction smoothing and with all corrections applied including point-spread function modeling. CT images were reconstructed using filtered back-projection (FBP) with 0.488 × 0.488 × 2.344 mm^3^ voxel size.

For SAFOV PET/CT imaging, the Siemens Biograph mCT scanner was utilized which combines a 128 slice CT scanner with a 21.8 cm PET FOV with multi-bed imaging implemented with a 43% overlap [23]. The CT imaging was acquired at 120 kVp using the CARE Dose 4D dose modulation with an average of 50–80 mAs. Images were reconstructed following the local clinical protocol. CT images were reconstructed with FBP and a 0.96 × 0.96 mm^2^ pixel size and 5.0 mm slice thickness. PET images were reconstructed with TOF enabled OSEM, 2 iterations, 21 subsets, 4.073 × 4.073 mm^2^ pixel size, 5.0 mm slice thickness, and a Gaussian smoothing with 2 mm slice thickness, along with all standard corrections including point-spread function modeling (TrueX).

### Image interpretation and semi-quantitative analysis

**Fig. 1:**
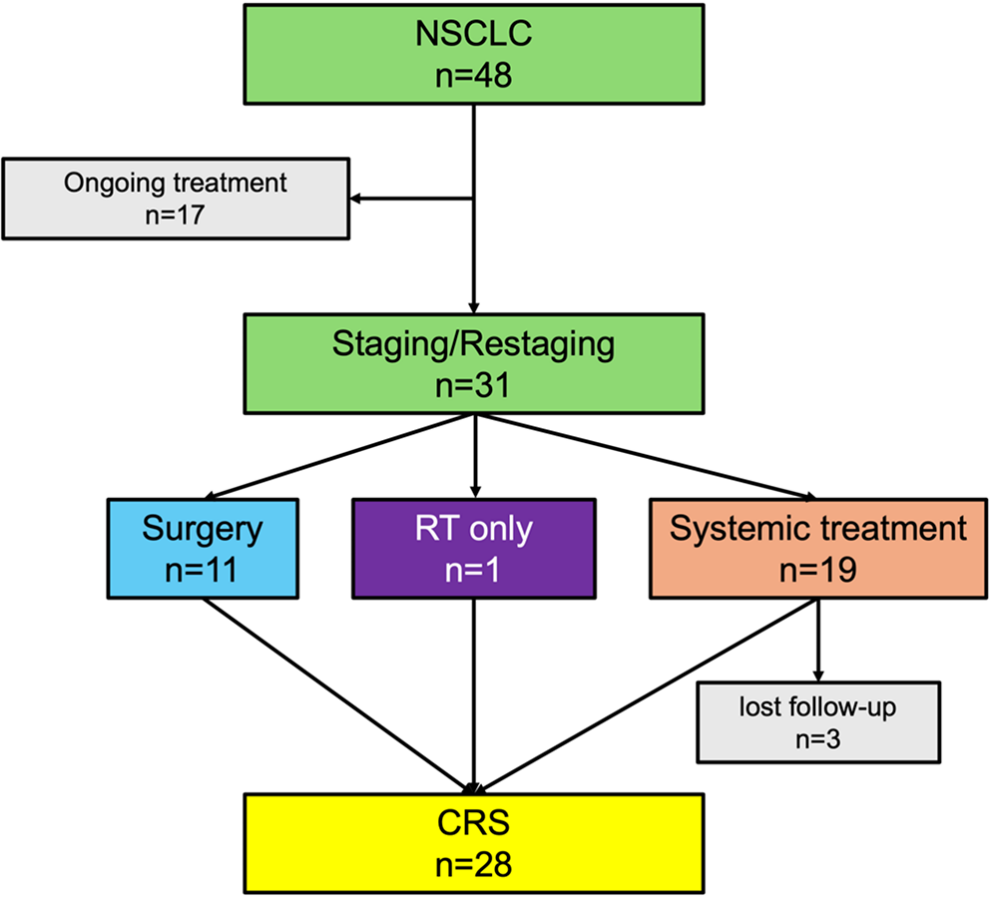
Study flowchart of all patients referred with non-small cell lung cancer (NSCLC). CRS = composite reference standard, RT = radiation therapy

Image interpretation was performed by 3 board certified nuclear medicine physicians. Both TB and SAFOV images were read in a randomized order with a one week pause between the reads. Lymph node levels (1R/L, 2R/L, 3a/p, 4R/L, 5, 6, 7, 8R/L, 9R/L, 10-14R/L) were assessed for the presence of [^18^F]FDG uptake binarily. TP, false positive (FP), true negative (TN) and FN lymph nodes were assessed per level with a composite reference standard (CRS) after a follow-up period of 24 month [range: 2–41 months] (*Supplementary Material*, Fig. 1). Nine patients died during the follow-up period. Correlative histopathology data per lymph node (by biopsy, fine needle aspiration or after surgery) was collected. Where no histopathology was available, treatment response and no response per lymph node level was assessed by the clinical interpretation of follow-up imaging (CT and PET/CT) after scrutiny of the patients’ charts. Where no treatment was performed, follow-up imaging data after active surveillance were used to assess CRS for each lymph node level [24]. Three patients without sufficient follow-up data were excluded from the analysis. Semi-quantitative lesion analysis was performed using a 40% iso-contour volume-of-interest (VOI) approach. The signal-to-noise ratio (SNR) was defined as the reciprocal coefficient of variation of the background where σ is the standard deviation (SD) of the background VOI and µ is the SUV_mean_ of the background VOI placed in patients’ right liver lobe [15]. Visually identifiable lymph nodes meaning that they could be distinguished from the surrounding background by increase uptake were quantified accordingly. Tumor-to-background ratio (TBR) was defined as previously published [25, 26]. For semi-quantitative image analysis, OsiriX MD version 13.0 (Pixmeo SARL, Switzerland) was used.

$$\:\text{S}\text{N}\text{R}=\frac{1}{CoV}=\left(\frac{\mu\:}{\sigma\:}\right),\:\text{T}\text{B}\text{R}=\frac{SUVmax\left(tumor\right)}{SUVmean\:\left(liver\right)}$$

### Statistical analysis

Statistical analysis was performed using Graphpad Prism Version 10 (San Diego, California) [27]. Diagnostic performance indices are presented with 95% confidence interval (CI) and were compared using McNemar’s test for sensitivity and specificity and permutation test for positive (PPV) and negative predictive values (NPV) [28]. Semi-quantitative data are presented as mean ± SD or as median and range. Paired t-tests after checking for normality were used to compare TB and SAFOV PET/CT image metrics. Receiver operating characteristics (ROC) and areas under the curve (AUC) were calculated to determine thresholds for the TB and SAFOV PET data. Thresholds were identified as the one achieving a minimum false positive rate by which sensitivity was > 80%. Comparisons yielding p-values less than 0.05 were considered statistically significant.

## Results

Of the 48 patients, who met the inclusion criteria 31 patients were referred for staging or restaging of NSCLC to our center. *N* = 11 patients received surgery after staging PET, one patient received localized radiation therapy and *n* = 19 patients received systemic treatment (adjuvant or neo-adjuvant) Fig. 1. CRS to confirm TP and TN mediastinal lymph nodes was formulated for 28 of the 31 patients of which *n* = 12 were enrolled in study arm 1 and *n* = 16 in study arm 2. Overall, 700 lymph node levels (57 positive, 643 negative) could be analyzed and correlated to the CRS on TB and SAFOV PET/CT.

### Lymph node sensitivity, specificity, PPV and NPV for both scans

Sensitivity and PPV were slightly higher on TB compared to SAFOV PET/CT without statistical significance (Sensitivity: 86.0% vs. 77.2%, *p* = 0.23; PPV: 81.7% vs. 72.1%, *p* = 0.08). Specificity and NPV were comparable between both scanners (Specificity: 98.3% vs. 97.4%, *p* = 0.13; NPV: 98.6% vs. 98.0% *p* = 0.22). Positive likelihood ratio was higher for TB PET/CT (50.3 vs. 29.2) whereas negative likelihood ratio were comparable between TB and SAFOV PET/CT (0.1 vs. 0.2). Detailed diagnostic accuracy indices with 95% CI are outlined in Table 2.

**Table 2:** Diagnostic performance indices on NSCLC N-staging Total-Body (TB) and short-axial field-of-view (SAFOV) PET/CT with 95% confidence interval (CI)

	TB PET	SAFOV PET	*p*-value
Sensitivity	86.0% (CI: 77.0–95.0%)	77.2% (CI: 66.3–88.1%)	0.23
Specificity	98.3% (CI: 97.3–99.3%)	97.4% (CI: 96.1–98.6%)	0.13
Positive predictive value	81.7% (CI: 71.9–91.5%)	72.1% (CI: 60.9–83.4%)	0.08
Negative predictive value	98.6% (CI: 97.9–99.6%)	98.0% (CI: 96.9–99.1%)	0.22
Positive Likelihood ratio	50.3 (CI: 27.7–91.1)	29.2 (CI: 17.9–47.7)	-
Negative Likelihood ratio	0.1 (CI: 0.1–0.3)	0.2 (CI: 0.2–0.4)	-
Accuracy	97.4% (CI: 96.0-98.5%)	95.7% (CI: 93.9–97.1%)	-

Incorrect changes in N-stage were reported in *n* = 6/28 (21.4%) for SAFOV PET (3 up- and 3 downstagings) and in *n* = 1/28 (3.5%) for TB PET/CT (1 upstaging). This resulted in a change in stage group in 5/28 cases (17.9%) for SAFOV and 1/28 cases (3.5%) for TB PET/CT.

### Semi-quantitative evaluation of mediastinal lymph node levels

**Fig. 2:**
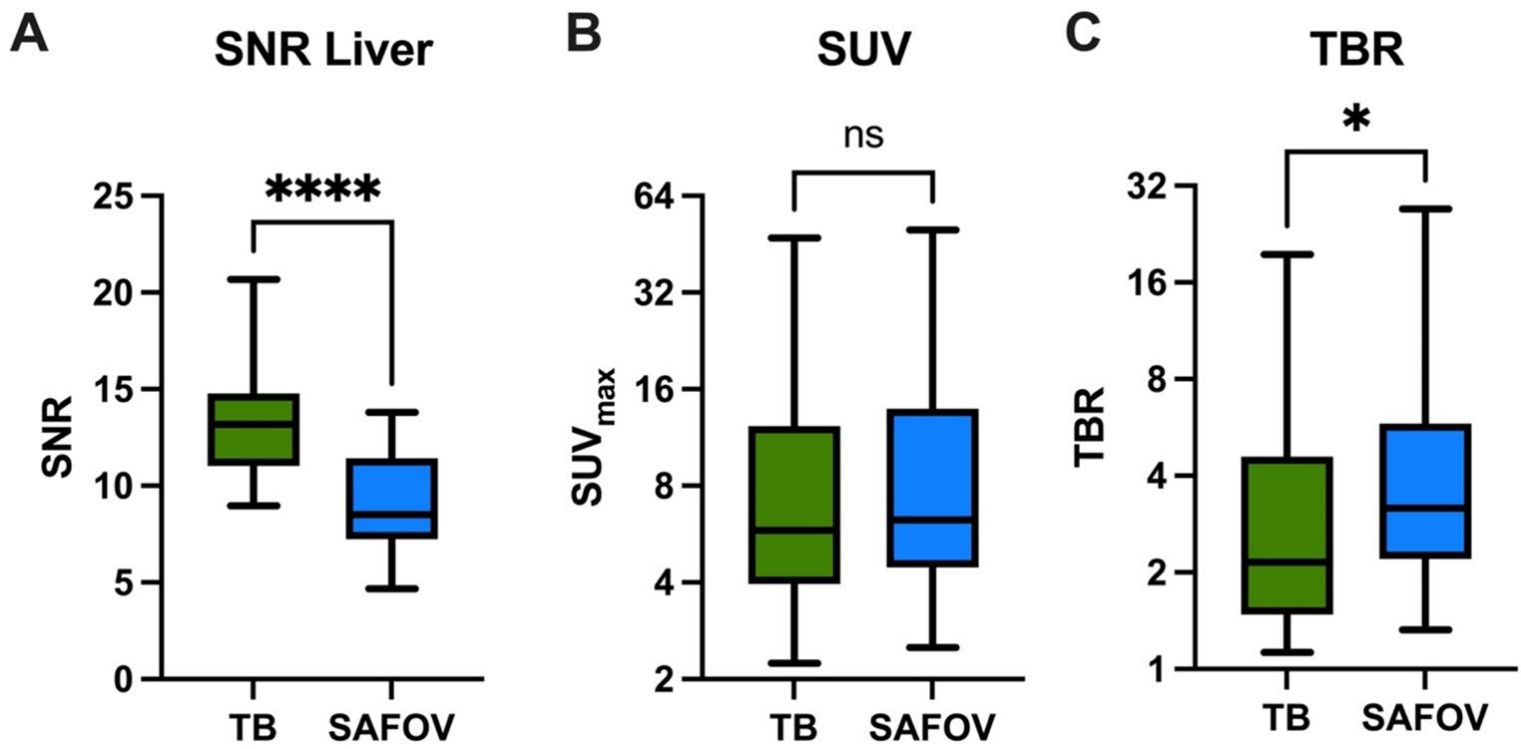
Semi-quantitative measurements of pathological lymph nodes on Total-Body (TB) and short-axial field-of-view (SAFOV) PET/CT. Displayed are signal-to-noise ratio (SNR, **A**), maximum standardized uptake values (SUV_max_, **B**) and tumor-to-background ratio (TBR, **C**). *p < 0.05, **p < 0.01, ***p < 0.001, ****p < 0.0001, ns = not statistically significant

Noise level was higher in SAFOV compared to TB PET/CT resulting in significantly higher SNR in TB PET (13.2±2.9 vs. 8.9±2.6, *p* < 0.01) (Fig. 2). Of 700 mediastinal lymph levels 10% were visually identifiable and quantifiable.

Lymph node tumor uptake for TP lymph node levels did not differ significantly between the scanners (SUV_max_ TB: 8.8±8.8 vs. SAFOV: 10.3±10.9, *p* = 0.48). However, TBR was significantly lower on TB compared to SAFOV PET (3.6±3.6 vs. 5.3±5.9, *p* = 0.04) indicating that, on average, TB PET/CT was able to successfully detect TP lymph nodes at a lower TBR threshold (Fig. 2 and *Supplementary Material*, Table 2).

There was no statistical difference for MTV (TB: 10.7±23.7 vs. SAFOV: 10.8±20.3, *p* = 0.45) and TLG (TB: 22.9±57.9 ca. 18.3±34.9, *p* = 0.91) for TP mediastinal lymph nodes between the scanners (*Supplementary Material*, Fig. 2).

### Receiver operating characteristics (ROC) for mediastinal lymph nodes

ROC analysis for the visually identifiable and quantifiable lymph nodes demonstrated equivalent or improved thresholds with TB PET. SUV_max_ thresholds were identified at 3.0 for both scanners. However, thresholds for TBR, MTV and TLG were lower on TB compared to SAFOV PET/CT.

TB PET/CT was able to detect mediastinal lymph node metastasis with 95.7% sensitivity and 83.9% specificity at a TBR of 1.2, whereas SAFOV PET/CT achieved comparable diagnostic accuracies (sensitivity: 84.2% and specificity: 82.9%) at TBR of 1.7. Nevertheless, AUC were comparable between TB (0.97) and SAFOV (0.93) PET/CT.

**Fig. 3:**
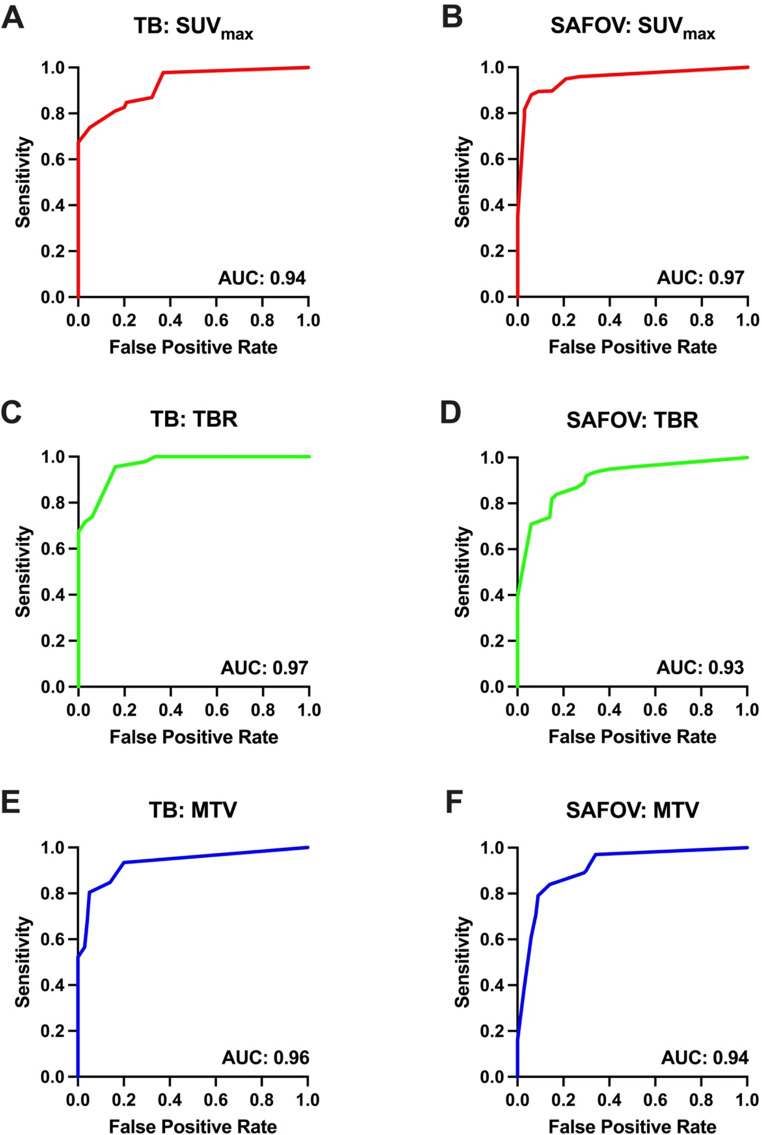
Receiver operating characteristics (ROC) of Total-Body (TB) and short-axial field-of-view (SAFOV) PET/CT. Displayed are maximum standardized uptake values (SUV_max_, **A**/**B**), tumor-to-background ratio (TBR, **C**/**D**) and metabolic tumor volume (MTV, **E**/**F**). AUC = area under the curve

For MTV TB PET/CT was able to detect lymph node metastasis at a threshold of 0.5 ml (sensitivity: 80.4%, specificity: 95.2%). The threshold of SAFOV PET/CT was MTV of 1.0 ml (sensitivity: 84.2%, specificity: 85.7%). AUC for MTV were comparable between both scanners (TB: 0.96 vs. 0.94) (Fig. 3 and *Supplementary Material*, Table 2).

For TLG similar results were found. TB PET threshold was 1.0 ml (sensitivity: 91.3%, specificity: 81.0%, AUC: 0.96) versus the SAFOV PET/CT threshold of 3.0 ml (sensitivity: 84.2%, specificity: 91.4%, AUC: 0.95) (*Supplementary Material*, Fig. 2).

## Discussion

In this prospective single center cross-over study we present the first data comparing TB and SAFOV PET/CTs’ diagnostic accuracy indices for N-staging with a head-to-head study design. We report high diagnostic accuracy indices for both scanners. Sensitivity and PPV in TB were slightly higher compared to SAFOV PET/CT without statistical significance.

Since its introduction [^18^F]FDG PET/CT is routinely used for staging and restaging of NSCLC patients [3]. SAFOV PET/CT has shown to be superior to CT chest in the detection of mediastinal lymph node metastases. Sensitivity and specificity for CT chest in the detection of mediastinal lymph node metastases (> 1 cm) were in the range of 51–64% and 74–86% [8, 29] and relied only on lymph node sizes [30]. Whereas sensitivity and specificity for SAFOV [^18^F]FDG PET/CT with 58–94% and 76–96% have been reported [7, 9, 31]. Moreover, a pooled metanalysis with 3028 patient data could specify the sensitivity and specificity to 72% and 90% for SAFOV PET [32]. Our data are in agreement with these values as we reported sensitivity and specificity of 77.2% and 97.4% for SAFOV PET/CT. Our SAFOV PET’s PPV of 72.1% and NPV of 98.0% were also in line with published data (PPV: 64% (CI: 43–80% and NPV: 95% (CI: 90–98%)) reported by Darling et al. in a prospective comparative trial with histopathological correlation of each mediastinal lymph node level [24].

Next-generation TB and LAFOV PET/CT systems have shown higher diagnostic accuracy, signal collection efficiency, and spatial resolution compared to analogue SAFOV PET/CT [11, 15, 33]. Consistent with these findings, our analysis revealed higher diagnostic accuracy in TB compared to SAFOV PET. TB PET’s sensitivity (86.0% vs. 77.2%, *p* = 0.23) was higher compared to SAFOV PET resulting in higher PPV (81.7% vs. 72.1%, *p* = 0.08); however without statistical significance. Thus, specificity (98.3% vs. 97.4%, *p* = 0.13) did not differ between both scanners resulting in comparable NPV (98.6% vs. 98.0%, *p* = 0.22) (Table 2).

**Fig. 4:**
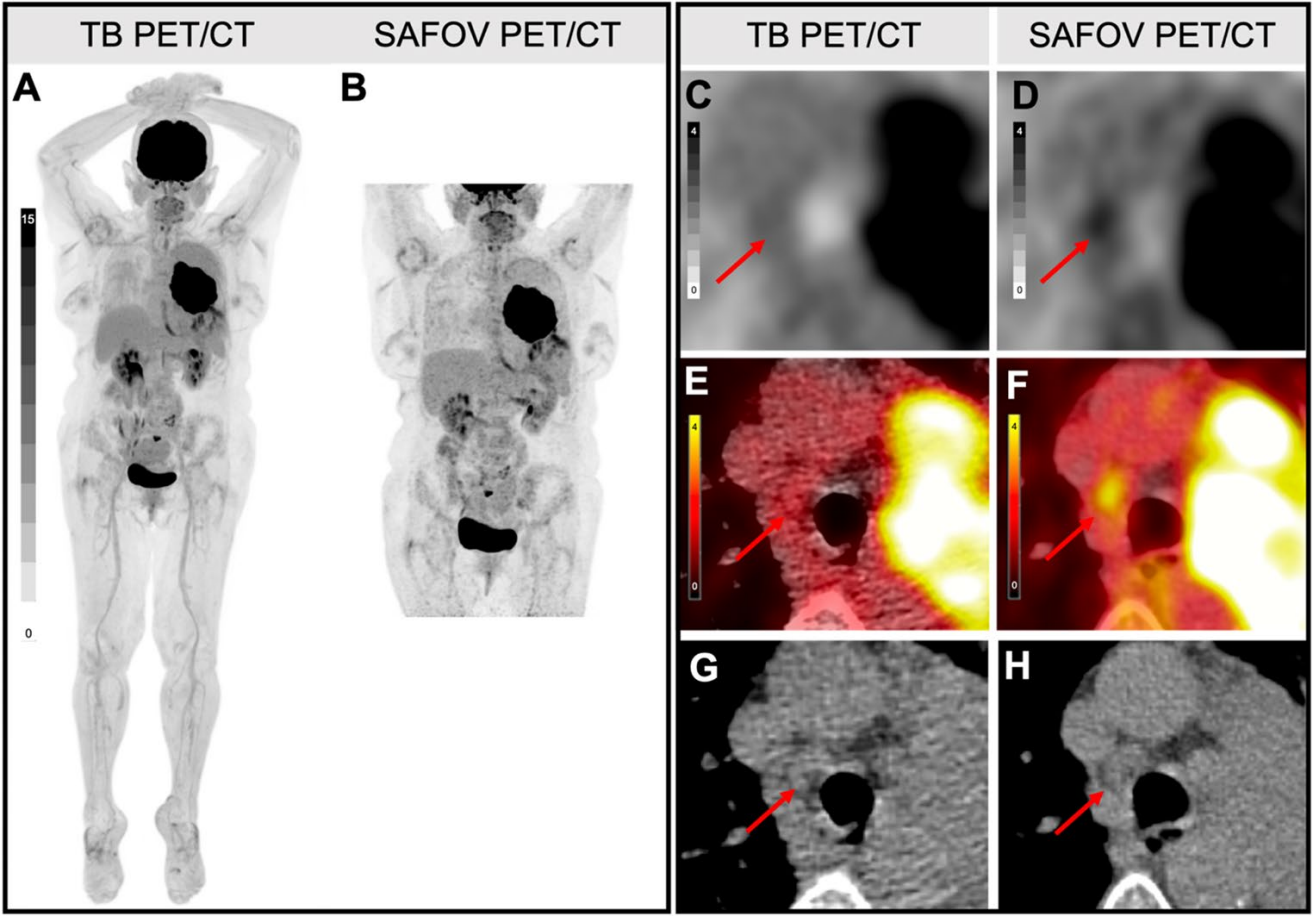
Example image of a lymph node (level 4R, marked with a red arrow), which was rated as positive on short-axial field-of-view (SAFOV) PET/CT and negative on Total-Body (TB) PET/CT. Composite reference standard (CRS) confirmed a negative lymph node. SAFOV PET/CT led to an incorrect upstaging to cN3. Tumor stage group was not affected. Displayed are maximum intensity projections (**A**/**B**), trans-axial PET-only (**C**/**D**), hybrid PET/CT (**E**/**F**) and CT-only (**G**/**H**) images

TB PET’s improved spatial resolution combined with higher sensitivity and signal collection efficacy might be a reason for the lower rate of FP and FN results (*Supplementary Material*, Table 1). Overall, NSCLC stage group was incorrect in 3.5% on TB and in 17.9% of SAFOV PET. Of note, the one case which was incorrectly upstaged on TB PET/CT was also incorrectly upstaged with SAFOV PET. Figure 4 shows an example of an incorrect upstaging in SAFOV PET, where TB PET was not mistaken. NCCN guideline recommends [^18^F]FDG PET/CT staging and restaging in all NSCLC tumor stage groups (I-IV). However, due to the lower PPV and higher FP-rate of SAFOV PET they also recommend to correlate PET-positive lymph nodes with histopathology to avert FP findings [4]. Our data for TB PET/CT showed higher PPV and lower FP-rate indicating that TB PET/CT might be a more robust diagnostic tool, which might help avoiding interventions and costs due to additional diagnostic test in the future.

Additionally, our data confirmed that noise statistics characterized by SNR were significantly lower on TB PET/CT compared to SAFOV PET (*p* < 0.01) [11, 15, 20]. Previous publications used the higher sensitivity of TB PET to instead reduce injected activities or shorten scan times [25, 34]. Moreover, our data indicate that lower noise characteristics might also be advantageous in detecting small and low [^18^F]FDG-avidity lesions [19]. TB PET’s improved signal collection efficiency for small lesions with low uptake was reflected in the semi-quantitative evaluation. ROC analysis revealed a SUV_max_ of 3.0 for both scanner as a threshold to identify a TP lesion, which is in agreement with previous reports [35]. However, TB PET/CT was able to detect TP lymph nodes at lower TBR (1.2 vs. 1.7) and smaller MTV (0.5 ml vs. 1.0 ml), which was reflected in the significantly lower TBR of TP mediastinal lymph nodes on TB compared to SAFOV PET (*p* = 0.04). Previous reports compared PET/CT with conventional CT chest staging and mainly focused on lymph nodes > 1 cm [9, 36]. Thus, our data supports its role in detecting small lymph node metastases at e.g. early cancer stages with a TB PET/CT MTV threshold of 0.5 ml, where TP lymph node metastases were detected with a sensitivity of 81% and a specificity of 95%. Nevertheless, there has been approaches to optimize SAFOV reconstruction characteristics to improve the detection of small lesions [37–39].

Figure 4 shows a 71 y/o female patient with NSCLC (subtype adenocarcinoma), who was referred to [^18^F]FDG PET/CT for staging. On SAFOV PET/CT a positive mediastinal lymph node (level 4R, SUV_max_: 2.8, TBR: 1.6, MTV: 0.4 ml, TLG: 1.3 ml) was detected. TB PET/CT did not show increased [^18^F]FDG uptake (SUV_max_: 2.0, TBR: 1.0, MTV: 0.3 ml, TLG: 0.9 ml) and was therefore rated as negative. CRS did not confirm a metastatic lesion resulting in a TN on TB PET and a FP on SAFOV PET finding, which lead to a correct change in N-staging on TB PET/CT (cN3 to cN2).

Some limitations of our study should be acknowledged. First, the analyzed sample size of 28 patients with CRS was small. However, our level-based mediastinal lymph node analysis with *n* = 700 levels, of which approximately 10% were visually identifiable and quantifiable, was comparable to other studies [3, 15, 24, 35]. Moreover, the head-to-head study design allowed us to minimize potential patient bias as the same patient was examined on both scanners at the same day. This design allowed also to minimize the radiation burden for the included patients as a second [^18^F]FDG injection was avoided. Secondly, uptake times varied as some patients were examined at 60- and other 90-minutes after the radiotracer injection depending on the randomization (study arm 1 vs. 2). To minimize bias due to different uptake times, a randomized cross over study protocol was established. The liver was identified as a reliable and reproducible reference organ for semi-quantitative analysis according to previous publications [21, 25]. Due to lost or incomplete follow-up there was an imbalance between the two study arms which could impact the results. Lastly, histopathological correlation was not possible for all assessed lymph nodes. Therefore, a CRS based on follow-up imaging after localized or systemic therapy was established to assess the true status of mediastinal lymph nodes [24, 40]. Our overall very long follow-up period (median: 24 month) could mitigate this limitation.

## Conclusion

In this small cohort, [^18^F]FDG TB and SAFOV PET/CT showed high diagnostic accuracy for N-staging in NSCLC patients. Sensitivity and PPV were slightly higher on TB PET without statistical significance. However, N-staging was more often correct on TB PET/CT and semi-quantitative thresholds to detect mediastinal lymph nodes were lower compared to SAFOV PET/CT. TB PET/CT might therefore be advantageous in finding small and low [^18^F]FDG-avid mediastinal lymph node metastases. However, SAFOV PET/CT might still be a useful tool in the diagnostic workup of N-staging in NSCLC with optimal reconstruction protocols.

## Electronic supplementary material

Below is the link to the electronic supplementary material.

Supplementary Material 1

## Data Availability

Data are available at the corresponding author’s address upon reasonable request.
