# Supplementary material for: Diagnostic accuracy in NSCLC lymph node staging with Total-Body and conventional PET/CT
Source: Eur J Nucl Med Mol Imaging. 2025 Mar 21;52(9):3190–8. doi: 10.1007/s00259-025-07177-3 (PMC12222388; doi:10.1007/s00259-025-07177-3)
Supplement: Supplementary file 1 — Supplementary Material 1 [file 259_2025_7177_MOESM1_ESM.docx]

# Supplementary Material

Table 1: Confusion matrix for Total-Body (TB) and short-axial field-of-view (SAFOV) PET/CT. Positive and negative results from both tests as well as from the composite reference standard (CRS) for N-staging of the enrolled patients with NSCLC.

| CRS | TB PET/CT | | SAFOV PET/CT | |
| --- | --- | --- | --- | --- |
|  | positive | negative | positive | negative |
| positive | 49 | 8 | 44 | 13 |
| negative | 11 | 632 | 17 | 626 |

Table 2: Maximum standardized uptake value (SUV_max_), tumor-to-background ratio (TBR), metabolic tumor volume (MTV) and total lesion glycolysis (TLG) thresholds for Total-Body (TB) and short-axial field-of-view (SAFOV) PET/CT.

|  | TB | | | | SAFOV | | | |
| --- | --- | --- | --- | --- | --- | --- | --- | --- |
|  | Threshold | Sensitivity | | Specificity | Threshold | Sensitivity | | Specificity |
| SUV_max_ | 3.0 | | 81% | 84% | 3.0 | | 90% | 85% |
| TBR | 1.2 | | 96% | 84% | 1.7 | | 84% | 83% |
| MTV (ml) | 0.5 | | 81% | 95% | 1.0 | | 84% | 86% |
| TLG (ml) | 1.0 | | 91% | 81% | 3.0 | | 84% | 91% |


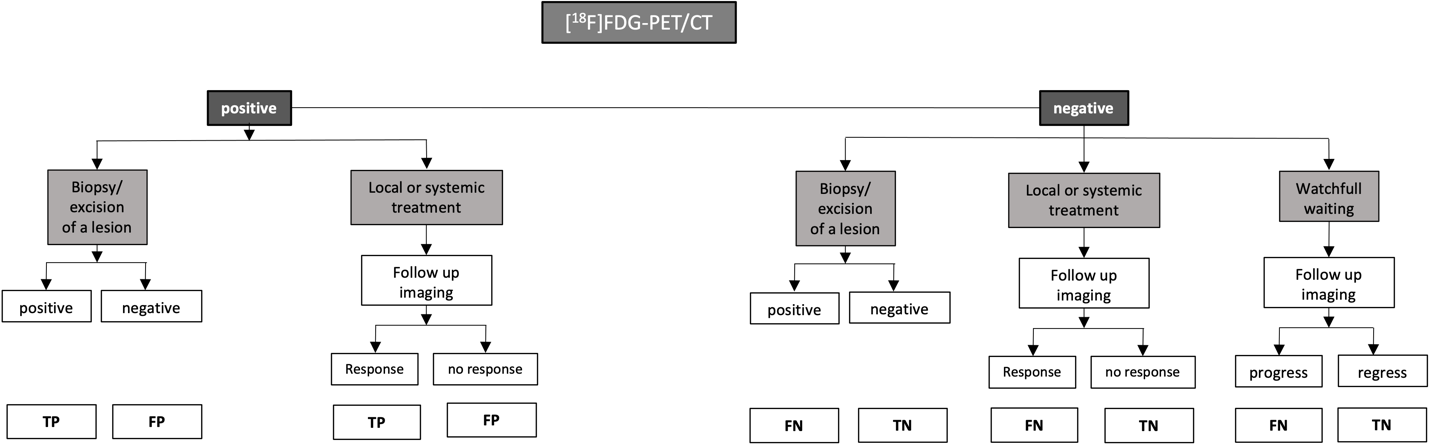


Figure 1: Flow chart to establish the composite reference standard (CRS) for each PET-positive mediastinal lymph node. TP=true positive, FP=false positive, TN=true negative and FN=false negative.


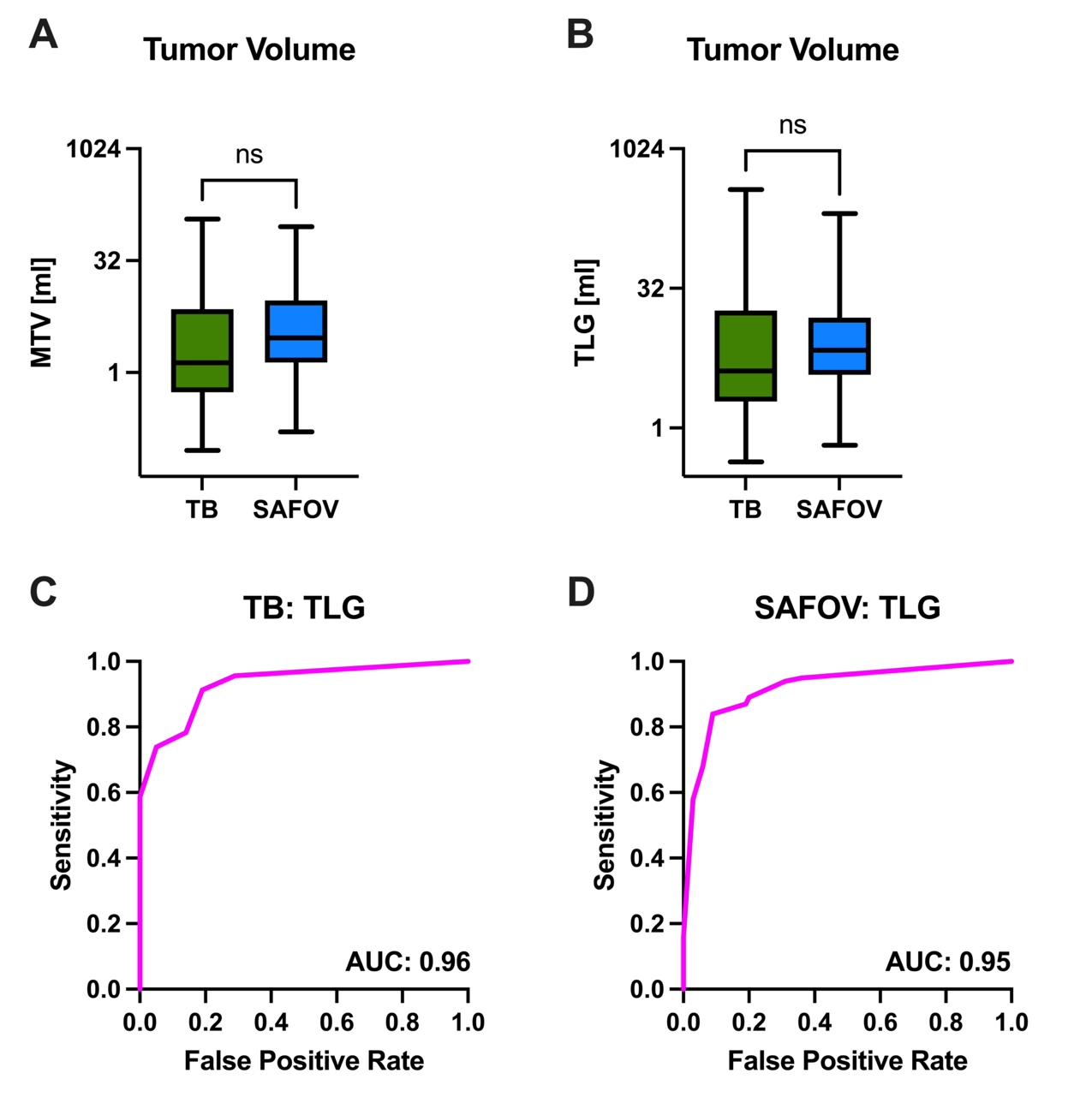


Figure 2: Displayed are metabolic tumor volumes (MTV, A) and total lesion glycolysis (TLG, B) on Total-Body (TB) and short-axial field-of-view (SAFOV) PET/CT. C and D show TLG receiver operator characteristics (ROC) for both scanners.
